# The Role of H2-Calponin Antigen in Cancer Metastasis: Presence of Autoantibodies in Liver Cancer Patients

**DOI:** 10.3390/ijms24129864

**Published:** 2023-06-07

**Authors:** Xiaoyun Bin, Yu Luo, Zefeng Sun, Chaoqun Lin, Peng Huang, Zhenbo Tu, Ling Li, Cong Qu, Jiamin Long, Sufang Zhou

**Affiliations:** 1 Department of Biochemistry and Molecular Biology, School of Basic Medical Sciences, Guangxi Medical University, Nanning 530021, China; 2 School of Basic Medical Sciences, Youjiang Medical University for Nationalities, Baise 533000, China; 3 Key Laboratory of the Ministry of Education Project for Early Prevention and Treatment of Regional High-Risk Tumors & Key Laboratory of Biological Molecular Medicine Research, Education Department of Guangxi Zhuang Autonomous Region, Guangxi Medical University, Nanning 530021, China

**Keywords:** CNN2, HCC, SEREX, serum markers, tumor metastasis

## Abstract

To investigate the potential of H2-calponin (CNN2) as a serum biomarker for hepatocellular carcinoma (HCC), this study employed the serological analysis of recombinantly expressed cDNA clone (SEREX) technique to identify the presence of CNN2 antibody in the serum of patients with HCC and other tumors. The CNN2 protein was produced through genetic engineering and used as an antigen to determine the positive rate of serum CNN2 autoantibodies via indirect enzyme-linked immunosorbent assay (ELISA). In addition, the mRNA and protein expressions of CNN2 in cells and tissues were evaluated using RT-PCR, in situ RT-PCR, and immunohistochemistry methods. The HCC group exhibited a significantly higher positive rate of anti-CNN2 antibody (54.8%) compared to gastric cancer (6.5%), lung cancer (3.2%), rectal cancer (9.7%), hepatitis (3.2%), liver cirrhosis (3.2%), and normal tissues (3.1%). The positive rates of CNN2 mRNA in HCC with metastasis, non-metastatic HCC, lung cancer, gastric cancer, nasopharyngeal cancer, liver cirrhosis, and hepatitis were 56.67%, 41.67%, 17.5%, 10.0%, 20.0%, 53.13%, and 41.67%, respectively. Meanwhile, the positive rates of CNN2 protein were 63.33%, 37.5%, 17.5%, 27.5%, 45%, 31.25%, and 20.83%, respectively. The down-regulation of CNN2 could inhibit the migration and invasion of liver cancer cells. CNN2 is a newly identified HCC-associated antigen that is implicated in the migration and invasion of liver cancer cells, making it a promising target for liver cancer therapy.

## 1. Introduction

Hepatocellular carcinoma (HCC) is a malignant tumor with high morbidity and mortality worldwide, especially in Asia [1]. Despite decreasing global tumor mortality, the mortality of HCC is on the rise [2,3,4]. This is due to the fact that most cases of HCC are diagnosed at an advanced stage, leaving little opportunity for surgical resection, and resulting in poor efficacy of chemotherapy or radiotherapy. Effective early diagnostic markers and therapeutic targets are therefore necessary to be explored for HCC.

Alpha-fetoprotein (AFP) is currently the most commonly used serological marker for diagnosing HCC [5]. However, up to 18–20% of primary HCC patients, especially elderly patients, have normal serum AFP levels [6], making HCC screening with AFP prone to missed detection or false positives. It is therefore necessary to identify additional HCC antigens and combine them with AFP to improve the detection rate and reduce the false positive rate. Calponin is a family of actin motion-related proteins expressed in smooth and non-smooth muscle cells. There are three subtypes of calponin in vertebrates—H1-calponin (CNN1), H2-calponin (CNN2), and H3-calponin (CNN3). The CNN2 gene is located on chromosome 19q13 with a molecular weight of 34 KDa. CNN2 plays a role in inhibiting smooth muscle contraction, participating in signal transduction, and maintaining skeleton stability by binding actin or interacting with a calcium-binding protein and auxiliary actin [7].

Recent research indicates that CNN2 plays a role in the proliferation, differentiation, and migration of tumor cells [8,9]. For instance, the down-regulation of CNN2 can significantly promote the proliferation and metastasis of prostate cancer cells [10]. When the CNN2 gene was knocked out in experimental mice, the motility and phagocytosis of macrophages were significantly enhanced, and they also exhibited a higher proliferation rate and faster mobility [8]. However, conflicting findings have shown that the knockout of endogenous CNN2 can activate caspase3 and caspase7 in gastric cancer cells, thereby suppressing the continued growth of cancer cells [11]. CNN2 has been found to be up-regulated in human breast cancer tissues but not in healthy and benign controls according to proteomic analysis [12]. It was also found that 10.5% of breast cancer patients had increased CNN2 expression in serum, suggesting that CNN2 can be used as a biomarker for the early diagnosis of breast cancer [13]. In our previous research, we used serological analysis of recombinantly expressed cDNA clone (SEREX) technology to screen the cDNA library of human undifferentiated embryonic stem cells with mixed serum from HCC patients and identified 30 cDNA clones that reacted positively with the serum antibody of HCC. After performing DNA sequencing and bioinformatics analysis on the 30 positive clones, we conducted additional screening using SEREX technology to detect high titers of anti-CNN2 autoantibodies in the serum of HCC patients, suggesting that CNN2 may be a novel HCC-associated antigen [14,15]. However, the expression level and cellular distribution of CNN2 remain unclear, and the impact of its increased or decreased expression on tumor cells has yet to be studied. Therefore, investigating the expression level of CNN2 in tumor tissues and its correlation with tumor development will offer a new theoretical foundation for targeted therapy of HCC. In order to confirm the specificity of CNN2 autoantibodies in the serum of HCC patients and the correlation between the expression of CNN2 in HCC tissues and the proliferation and metastasis of HCC cells, we employed SEREX technology to detect CNN2 autoantibodies in the serum of HCC patients, hepatitis patients, and patients with other cancers. Additionally, we utilized ELISA to validate the level of CNN2 autoantibodies in the blood of HCC patients. We also analyzed the expression levels of CNN2 in tumor cell lines and tissues to explore its potential as a molecular biomarker for HCC.

## 2. Results

### 2.1. CNN2 Autoantibodies Exist in the Serum of Some HCC Patients

**Figure 1:**
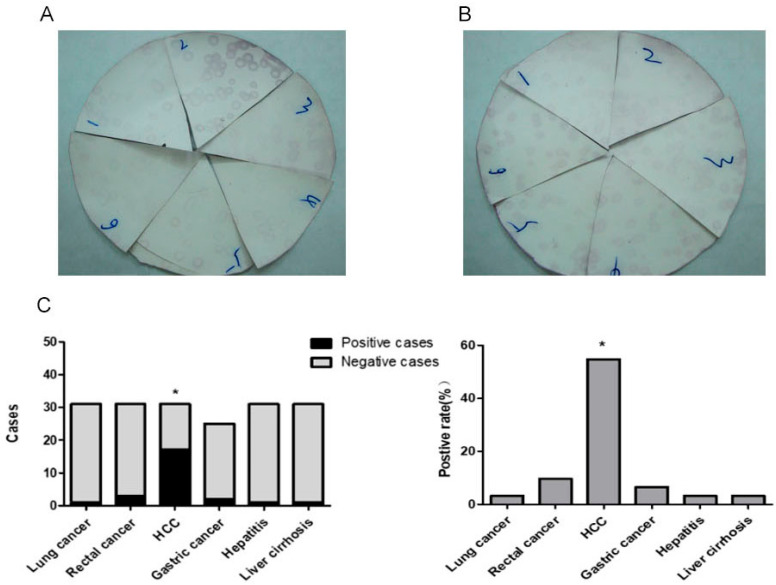
Detection of anti-CNN2 antibodies in serum of different patients by SEREX technology. (**A**) Plaques transfected with CNN2-λ-ZAP. (**B**) Plaques transfected with λ-ZAP. 1: gastric cancer; 2: HCC; 3: hepatitis; 4: lung cancer; 5: rectal cancer; 6: liver cirrhosis. (**C**) Expression statistics of anti-CNN2 antibody in serum of different patients. * *p* < 0.05.

Serum samples from patients with HCC, gastric cancer, lung cancer, colorectal cancer, hepatitis, or cirrhosis (31 cases each) and healthy controls (32 cases) were analyzed using the SEREX technique.XL1-Blue MRF’ *Escherichia coli* was transfected with CNN2-λ-ZAP and λ-ZAP, respectively, and cultured in LB solid medium to produce plaque. The plaque on NC membrane was western blot and compared. The plaques that expressed CNN2 appeared darker in color and were identified as positive when compared to the empty plaques (Figure 1A,B). Our results show that the positive rate of CNN2 antibody in the HCC patients was 54.8%, which was significantly higher than that in the gastric cancer (6.5%), lung cancer (3.2%), rectal cancer (9.7%), hepatitis (3.2%), cirrhosis (3.2%), and normal tissue samples (3.1%) (*p* < 0.05) (Table 1) (Figure 1C). Furthermore, there was no statistically significant correlation between the positive rate of CNN2 antigen and clinical indicators of HCC patients, such as age, AFP value, tumor pathological grade, and γ-glutamyltranspeptidase (GGT) (Supplementary Table S1).

### 2.2. CNN2 Recombinant Protein Was Successfully Purified

**Figure 2:**
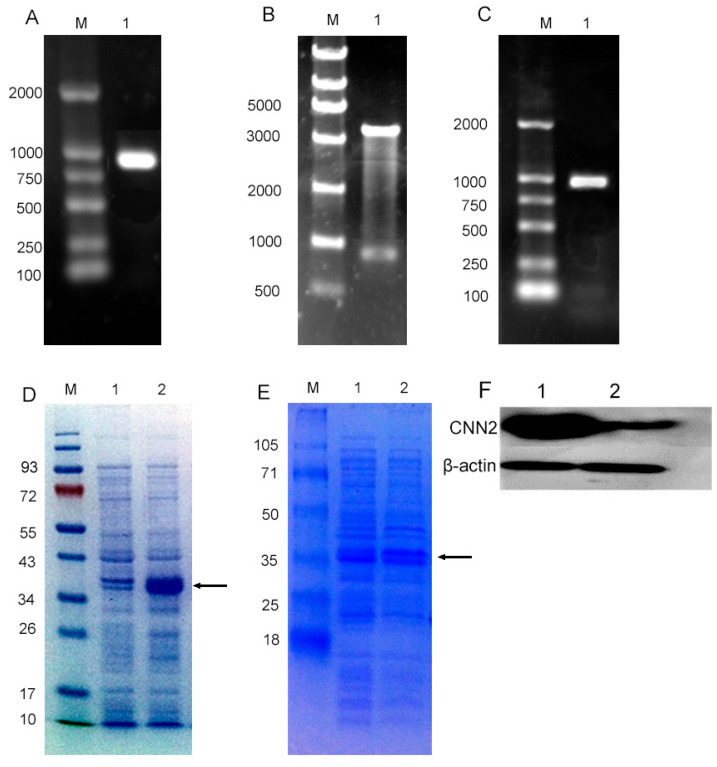
Identification of recombinant H2-calponin protein and detection of H2-calponin immunogenicity. (**A**) PCR amplification of CNN2 gene. Column M: marker; Column 1: CNN2 PCR products. (**B**) Plasmid identification by double enzyme digestion. Column M: marker; Column 1: pCold III-CNN2 digested by Nde I and Sal I. (**C**) PCR identification of CNN2 gene in genetically engineered bacteria. Column M: marker; Column 1: PCR products amplified from pCold III-CNN2. (**D**) Identification of CNN2 protein in genetically engineered bacteria. (**E**) Identification of soluble expression of CNN2 protein. Column M: protein marker; Column 1: before adding IPTG; Column 2: induction with 0.6 mM IPTG; arrows indicate CNN2 protein. (**F**) WB detection of CNN2 protein expression. Column 1: insoluble fragment; Column 2: soluble fragment. Arrows represented target protein.

The CNN2 gene was amplified by PCR using the CNN2 gene recombinant phage as a template. Agarose gel electrophoresis revealed a product of approximately 1000 bp in size, which is consistent with the expected gene fragment size of 969 bp (Figure 2A). The PCR product was recovered and digested with *Nde* I and *Sal* I enzymes, and then linked with the pCold III plasmid. The recombinant plasmid pCold III-CNN2 was transformed into *DH5α* and amplified. The pCold III-CNN2 plasmid was confirmed by enzyme digestion. The size of the fragment was found to be consistent with the target gene (Figure 2B). After sequencing, the pCold III-CNN2 recombinant plasmid was found to be completely consistent with the CNN2 sequence alignment on NCBI. The recombinant plasmid pCold III-CNN2 was transformed into *E. coli* BL21 (DE3)pLysS, and the genetically engineered bacteria were verified by antibiotic screening and PCR. The PCR results show that the target gene fragment was observed at about 1000 bp by agarose gel electrophoresis, as expected (Figure 2C). The targeted protein was observed by SDS-PAGE electrophoresis at approximately 34 kDa, which is in agreement with the anticipated size (Figure 2D). The targeted protein was detected in both bacterial protein and supernatant (Figure 2E). The purified His-CNN2 fusion protein could be detected by Western blot with anti-CNN2 antibody, indicating that the protein retained its immunogenicity (Figure 2F).

### 2.3. Detection of Serum CNN2 Antibody by Indirect ELISA

**Figure 3:**
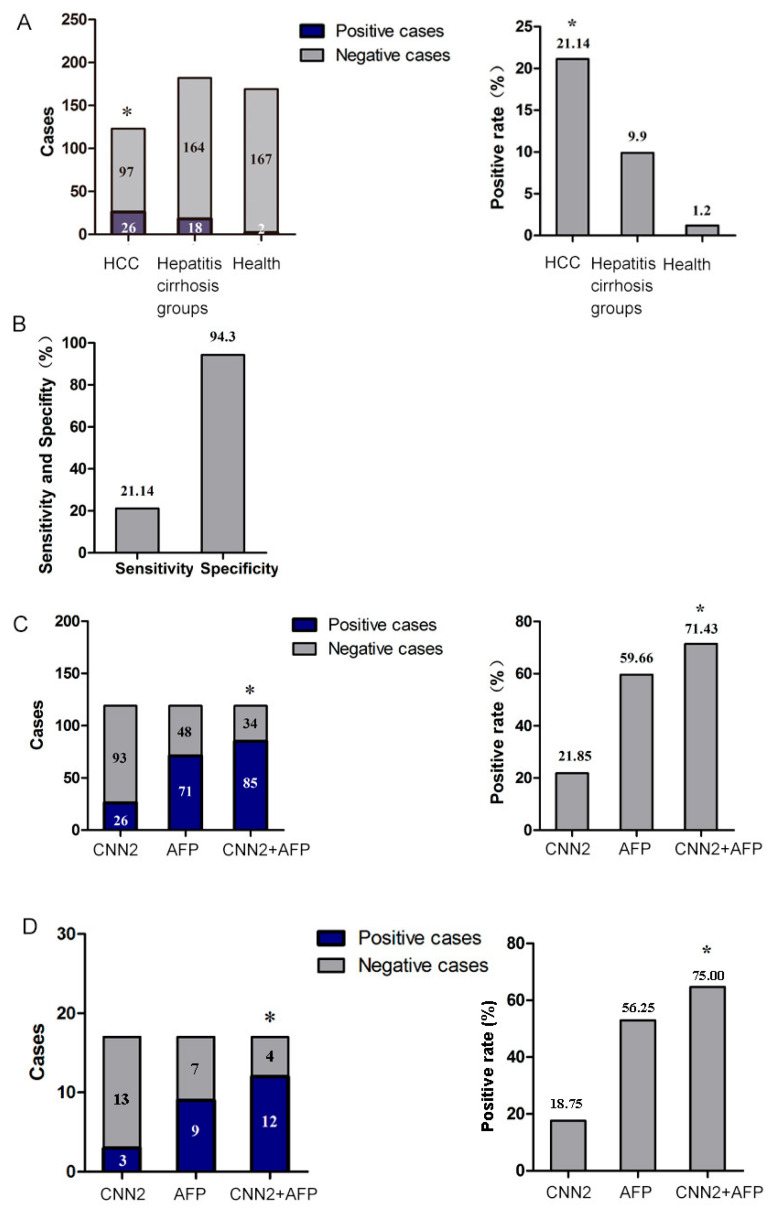
Detection of serum CNN2 antibody by indirect ELISA. (**A**) Positive rate of serum antibodies against CNN2 in HCC, hepatitis cirrhosis, and healthy group. (**B**) The sensitivity and specificity of anti-CNN2 antibodies. (**C**) The positive rate of HCC detected by AFP and CNN2 combination. (**D**) The positive rate of small HCC detected by AFP and CNN2 combination. * *p* < 0.05.

We coated the ELISA plate with the purified CNN2 recombinant protein and detected the presence of CNN2 antibody in the serum of patients with primary HCC, hepatitis cirrhosis, and normal tissue by the indirect ELISA method. According to the standard of the positive value (cut-off value) defined by normal tissue $\;\bar{x}\;$± 3sd, 26 positive cases were identified from 123 serum samples of HCC, and the positive rate was 21.14%. In addition, 18 of 182 positive cases of hepatitis and cirrhosis were detected. The positive rate was 9.9%, and 2 of the 169 normal human sera were positive, with a positive rate of 1.2% (Figure 3A). No statistically significant correlation was found between the positive rate of CNN2 and clinical indicators in the liver cancer case group. This means that the positive detection of anti-CNN2 antibody in the serum cannot be attributed to factors such as age, gender, AFP value, pathological grade, presence of cirrhosis, ALT value, AST value, HBsAg, and tumor size (Supplementary Table S2). The diagnostic value of CNN2 was evaluated using receiver operating characteristic (ROC) curve analysis. The area under the ROC curve was 0.73, indicating that CNN2 had a moderate diagnostic value in the HCC group. The sensitivity and specificity of CNN2 were 21.14% and 94.30%, respectively (Figure 3B). The combined detection of CNN2 and AFP for HCC yielded a positive rate of 71.43%, which was significantly higher than the positive rate of single detection with CNN2 (21.85%) and AFP (59.66%) (*p* < 0.05) (Figure 3C). Notably, the combination of the CNN2 and AFP detection methods exhibited significant advantages in detecting small HCC. Out of 16 small HCC samples, the CNN2 detection rate was 18.75%, while the AFP detection rate was 56.25%. However, when the CNN2 and AFP detection methods were combined, the positive rate increased to 75.00%, which was significantly higher than that of either single detection method (*p* < 0.05) (Figure 3D).

### 2.4. CNN2 Was Highly Expressed in HCC Tissues and Cells

**Figure 4:**
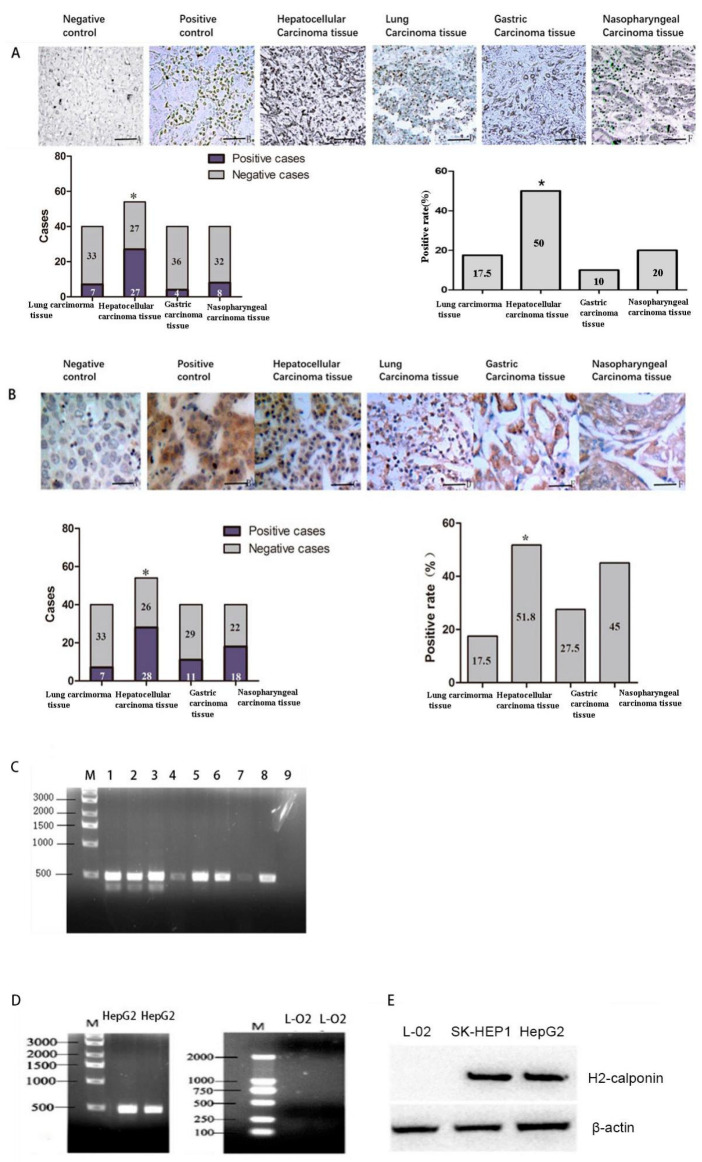
CNN2 was highly expressed in HCC tissues and cells. (**A**) Expression of CNN2 mRNA detected by in situ RT-PCR in negative control, positive control, and 4 cancerous tissues (×100) (* *p* < 0.05). (**B**) Expression of CNN2 protein detected by immunohistochemistry in negative control, positive control, and 4 cancerous tissues (×400) (* *p* < 0.05). (**C**) Expression of CNN2 mRNA expression in tumor cell lines detected by RT-PCR. Columns 1, 2: gastric cancer; Columns 3, 8: lung cancer; Columns 4, 7: breast cancer; Columns 5, 6: HCC; Column 9: normal liver cell. (**D**) Expression of CNN2 mRNA in HCC and normal hepatocytes. (**E**) Expression of CNN2 protein in HCC and normal hepatocytes.

In situ RT-PCR detection showed that CNN2 mRNA was mainly distributed around the nucleus. The positive rate of CNN2 mRNA expression in lung cancer, HCC, gastric cancer, and nasopharyngeal cancer was 17.5% (7/40 cases), 50.0% (27/54 cases), 10.0% (4/40 cases), and 20.0% (8/40 cases), respectively (Figure 4A). The positive rate of CNN2 mRNA in HCC was significantly higher than that in other tumor tissues (*p* < 0.05).

Immunohistochemical analysis showed that CNN2 protein was mainly distributed in the cytoplasm with occasional nuclear staining. The positive rate of CNN2 protein expression in lung cancer, HCC, gastric cancer, and nasopharyngeal carcinoma was 17.5% (7/40 cases), 51.8% (28/54 cases), 27.5% (11/40 cases), and 45% (18/40 cases), respectively. The positive rate of CNN2 protein expression in HCC was significantly higher than that in other groups (*p* < 0.05) (Figure 4B). No significant difference was found in the positive rate of CNN2 among the lung cancer, gastric cancer, and nasopharyngeal carcinoma tissues. The HCC patients were grouped according to age, sex, pathological grade, and metastasis. The *χ*^2^ test showed that CNN2 was expressed in all HCC patients regardless of tumor grade. However, the positive rate of CNN2 expression in the HCC tissues with metastasis was significantly higher (63.33%) than that in the HCC tissues without metastasis (33.33%) (*p* < 0.05) (Supplementary Table S3).

**Figure 5:**
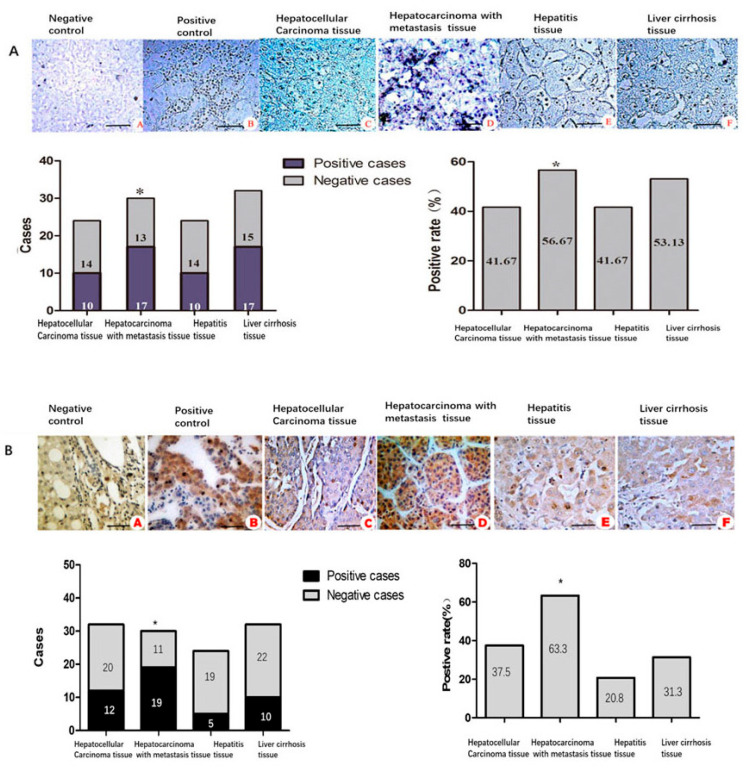
CNN2 was most highly expressed in HCC with metastasis. (**A**) In situ RT-PCR detection of CNN2 mRNA expressions in different liver diseases (×400). (**B**) Immunohistochemical detection of CNN2 protein expression in different liver diseases (×400). * *p* < 0.05.

RT-PCR was used to detect the mRNA expression of CNN2 in tumor cells, and strong expressions were amplified in the HCC, gastric cancer, and lung cancer cell lines. However, a weak amplification band appeared in the breast cancer cells, while in the normal human hepatocytes, no CNN2 mRNA expression was found (Figure 4C). There was no CNN2 mRNA or protein expression detected in the normal hepatocytes (L-O2) by the RT-PCR or Weston blot methods, while there were strong expression bands in the liver cancer cells (Figure 4D,E). Positive rates of CNN2 mRNA expression detected by in situ RT-PCR were 41.67% (10/24 cases) in non-metastasis HCC, 56.67% (17/30 cases) in HCC with metastasis, 41.67% (10/24 cases) in hepatitis and 53.13% (17/32 cases) in liver cirrhosis. The positive rate in HCC with metastasis tissues was the highest in all groups (*p* < 0.05) (Figure 5A). The positive rates of CNN2 protein expression detected by immunohistochemistry were 37.5% (12/32) in non-metastasis HCC, 63.3% (19/30) in HCC with metastasis, 20.8% (5/24) in hepatitis, and 31.3% (10/32) in liver cirrhosis, respectively. There was a statistically significant difference in the positive rate of CNN2 protein between HCC with metastasis and hepatitis, liver cirrhosis tissues (*p* < 0.05), but no significance was found between non-metastasis HCC, hepatitis, and liver cirrhosis tissues (*p* > 0.05) (Figure 5B).

### 2.5. Construction of CNN2-Silenced Liver Cancer Cell Lines by siRNA

**Figure 6:**
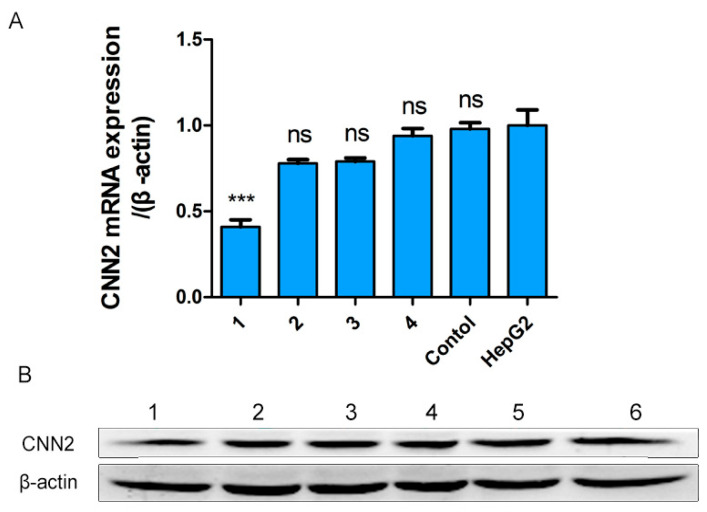
Fluorescence quantitative PCR and Western blot results of CNN2 siRNA. (**A**) Mean 2^−∆∆Ct^ value of qPCR results. *** *p* < 0.001 vs HepG2, ns *p* > 0.05 vs HepG2. Column 1: pGPU6-GFP-Neo-CNN2 siRNA1; Column 2: pGPU6-GFP-Neo- CNN2 siRNA2; Column 3: pGPU6-GFP-Neo-CNN2 siRNA3; Column 4: pGPU6-GFP-Neo-CNN2 siRNA4. (**B**) Image of Western blot. Column 1: pGPU6-GFP-Neo-CNN2 siRNA1; Column 2: pGPU6-GFP-Neo-CNN2 siRNA2; Column 3: pGPU6-GFP-Neo-CNN2 siRNA3; Column 4: pGPU6-GFP-Neo-CNN2 siRNA4; Column 5: pGPU6-GFP-Neo negative control, Column 6: HepG2 cells.

We employed fluorescence quantitative PCR to evaluate the efficacy of the four designed CNN2-siRNA sequences (siRNA1, siRNA2, siRNA3, siRNA4), and observed that all of them caused a reduction in CNN2 mRNA expression, with siRNA1 displaying the most significant effect compared to the control group (*p* < 0.05) (Figure 6A). Additionally, Western blot analysis indicated that siRNA1 had the most prominent inhibitory effect on CNN2 protein expression (Figure 6B) (*p* < 0.05).

### 2.6. Inhibition of CNN2 Can Reduce the Invasion and Migration of Liver Cancer Cells In Vitro

**Figure 7:**
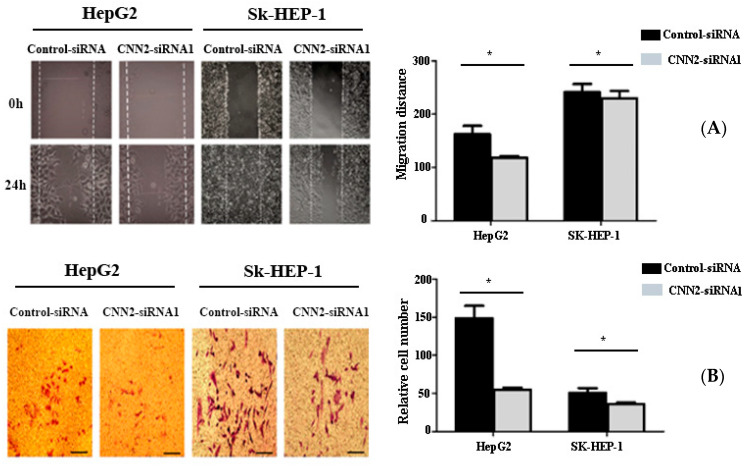
Down-regulation of CNN2 inhibits the migration and invasion of hepatocellular carcinoma cells. (**A**) Results of scratch healing experiment. (**B**) Results of Transwell Matrigel assay. * *p* < 0.05.

We selected the siRNA1 interference sequence, which had the highest inhibitory effect, to construct CNN2-knockdown cell models. The motility of liver cancer cells was evaluated through a scratch-healing experiment after CNN2 silencing with CNN2-siRNA1. Our results indicate that the Sk-HEP-1/HepG2 cells in the control group exhibited healing after 24 h of scratch, whereas the healing ability of the CNN2-siRNA group was significantly reduced (Figure 7A). The scratch width between these two groups showed statistical significance (*p* < 0.05), suggesting that CNN2-siRNA was capable of inhibiting the motility and migration of liver cancer cells. The cell invasion assays revealed that the number of transmembrane cells in the CNN2-siRNA group was significantly lower than that of the control group (*p* < 0.05). This finding suggests that inhibiting the expression of CNN2 can reduce the invasion of HepG2 and SK-HEP1 cells (Figure 7B).

## 3. Discussion

Calponin is a family of proteins associated with actin motion, expressed in both smooth and non-smooth muscle cells. In vertebrates, there are three homologous genes—CNN1, CNN2, and CNN3, encoding 3 respective isoforms [7]. CNN1 is primarily expressed in differentiated smooth muscle cells and is involved in smooth muscle contraction. On the other hand, CNN2 is not only expressed in muscle cells but also detected in human keratinocytes, epithelial cells, fibroblasts, and other non-muscle cells [9]. The calponin family of proteins has promising potential in tumor therapy. For instance, bioinformatics analysis suggests that CNN1 can be a potential bladder cancer biomarker and therapeutic target [16]. CNN1 has been found to inhibit the invasion and migration of lung squamous carcinoma cells and prevent the transformation of epithelial to mesenchymal. Conversely, inhibiting the expression of CNN1 can promote breast cancer metastasis [17,18]. It is hypothesized that tumor-targeted therapy against CNN2 expression could be a promising approach for treating human tumors, and the knockdown of CNN2 inhibits cell proliferation, blocks cell cycle at the S-phase, inhibits cell invasion and migration, and inhibits xenotransplanted tumor growth in nude mice [19,20]. Our previous research also demonstrated that CNN2 plays a critical part in tumor growth and metastasis, and it may serve as a potential target for molecular-targeted therapy of liver cancer [21].

It is known that autoantibodies against tumor antigens can be detected in the serum of cancer patients, even in the early stages [22]. For instance, s-p53-Abs have been found to have a high positive rate in many types of cancer [11,23]. SEREX is a technique used to discover and identify tumor-associated antigens, which has been employed in the screening of various types of tumors, such as lymphoma, leukemia, esophageal cancer, and gastric cancer [24,25,26,27,28]. In this study, we utilized SEREX technology to examine the presence of CNN2 antibodies in the serum of individuals with HCC, gastric cancer, lung cancer, colorectal cancer, hepatitis, cirrhosis, and healthy individuals. Our findings show a significantly elevated expression of CNN2 in the serum of HCC patients, while no statistically significant difference was observed between the positive rate of CNN2 in the serum of other tumor patients and that of the healthy control group. Moreover, there was no significant correlation between the positive rate of the CNN2 antigen and the clinical indicators of HCC patients, such as age, AFP value, tumor pathological grade, and GGT. These results suggest that SEREX technology can effectively screen CNN2 as an HCC-associated antigen, and CNN2 is closely associated with the occurrence and progression of HCC, but not with its deterioration. In a similar study, SEREX technology was used to screen autoantibodies in breast cancer, with 70% sensitivity and 91% specificity [29]. Kobayashi et al. [30] also reported that in patients diagnosed with cancers of the digestive organs, including esophageal squamous cell carcinoma, gastric cancer, and colon cancer, of the 14 antigens identified, 10 were potential diagnostic tools for esophageal squamous cell carcinoma, 8 for gastric cancer, and 3 for colon cancer. Their antibody levels were significantly higher than those of healthy donors detected by SEREX.

The primary method for screening autoantibodies against tumor-associated antigens in the serum of tumor patients is SEREX technology, but its complexity makes it difficult to use for large sample sizes. To investigate the potential of CNN2 for diagnosing, treating, and predicting the prognosis of HCC, we employed an indirect ELISA method using recombinant CNN2 protein-coated ELISA plates to detect anti-CNN2 antibodies in the serum from patients with primary liver cancer, hepatitis cirrhosis, and normal controls. The results indicate that the positive rate of anti-CNN2 antibodies in the HCC patients’ serum was significantly higher than that in the patients with cirrhosis or the normal controls. According to the ROC analysis, CNN2 exhibited diagnostic potential, with a sensitivity of 21.14% and specificity of 94.30%. However, the positive rate of serum CNN2 antibody in HCC was not related to clinical indicators such as age, sex, AFP value, BCLC stage, cirrhosis, ALT value, AST value, HBsAg, tumor size, etc. These results are consistent with our SEREX research results.

The AFP, a vital tumor marker for HCC detection, has limited sensitivity in identifying early and small HCC [31,32,33]. As a result, some researchers have attempted to combine AFP with other HCC-related factors to improve detection efficiency, and they have achieved promising results. For instance, AFP combined with PIVKA-II [34], AFP-L3, and DCP [35] and AFP with GP73 and DCP [36,37] have all been found to enhance the diagnostic value of AFP for HCC. According to the research, a group of autoantibodies against tumor-associated antigens, such as Sui1, p62, RalA, p53, NY-ESO-1, and c-myc, had an additive effect with AFP in detecting HCC [38]. Our study also shows that combining AFP and CNN2 increased the detection rate of HCC and small HCC, with positive detection rates of 71.43% and 75.00%, respectively. Thus, CNN2 could potentially serve as a useful clinical diagnostic tool to assist in detecting HCC.

We found that CNN2 was highly expressed in liver cancer cell lines, but not in normal liver cell lines. In addition, our study also found that the mRNA and protein expression levels of CNN2 were significantly higher in HCC tissues than in other tumor tissues, but there was no significant difference in the expression levels in non-metastatic HCC, hepatitis, and cirrhosis. However, the difference in the positive expression rate of CNN2 mRNA and protein is statistically significant in metastatic HCC and non-metastatic HCC. All of our research shows that the serum antibody titers and tissue mRNA and the protein expression levels of CNN2 in HCC patients had abnormal expression, especially in metastatic HCC. When we silenced CNN2 expression in HepG2 cells, the cell scratch and cell migration assay showed that suppressing the expression of CNN2 significantly hindered the migration and invasion of liver cancer cells. Similarly, there have also been studies showing that the expression of CNN2 is related to the invasion ability of colon cancer, and a high level of CNN2 expression has been detected in colon cancer tissues [22]. Based on these findings, we speculate that CNN2 might be an antigen associated with HCC, and its expression intensity could be related to the degree of metastasis. Therefore, CNN2 may be used as a biomarker of HCC rather than a marker of other liver cancer diseases.

The mechanism underlying CNN2’s involvement in tumor metastasis is not yet fully understood. One of the most important aspects of tumor invasion and metastasis is the destruction of the extracellular matrix barrier [39]. It is widely accepted that tumor cells activate and secrete various proteolytic enzymes after adhering to different components of the matrix through their surface receptors [40]. However, it is unclear whether the down-regulation of CNN2 expression affects the adhesion of HCC cells to the extracellular matrix through protein-degrading enzymes, endothelial cell migration, and tumor angiogenesis, or via cytoskeletal remodeling and molecular adhesion involved in tumor metastasis. Further research is needed to explore and understand the underlying mechanisms.

## 4. Materials and Methods

### 4.1. Clinical Specimen

Serum samples from individuals with HCC, lung cancer, gastric cancer, rectal cancer, hepatitis, liver cirrhosis, and healthy individuals were collected at the First Affiliated Hospital of Guangxi Medical University and stored at a temperature of −80 °C.

Tumor tissues of HCC, lung cancer, gastric cancer, and nasopharyngeal carcinoma, as well as adjacent tissues with margins greater than 5 cm, were collected at the First Affiliated Hospital of Guangxi Medical University. In total, 54 HCC and 40 other tumor tissues, along with 8 normal controls for each group, were obtained within 30 min in vitro. The tissue samples were then sent to Xi’an Ailina Biotechnology Co., Ltd. (Xi’an, Shanxi, China) for tissue chip preparation (TMA) and freezing. All specimens were pathologically diagnosed, and the relevant clinical data were collected. The Ethics Committee of Guangxi Medical University (20200035) approved this study, and informed consent was obtained from all participating patients and healthy donors.

### 4.2. Cell Lines and Cell Culture

The Sk-hep-1, HepG2, and L-02 cell lines (Shanghai Institutes of Biological Sciences, Chinese Academy of Sciences, Shanghai, China) were maintained in Dulbecco’s Modified Eagle’s Medium (DMEM) (SH30243.01, Hyclone, Kentucky, UT, USA) supplemented with 10% fetal bovine serum (16000-044, Gibco, New York, NY, USA) and incubated at 37 °C in a 5% CO_2_ atmosphere. The culture medium was replaced every two days.

### 4.3. SEREX Detection of Anti-CNN2 Antibodies in the Blood of Tumor Patients

XL1-BlueMRF’ *E. coli* and XL1-BlueMRF’ *E. coli* transfected with CNN2-λ-ZAP phage were preserved in our laboratory [31].

The XL1-Blue MRF’ *E. coli* were harvested and underwent repeated freeze–thaw cycles, followed by ultrasonic cracking to obtain the bacterial lysate. To avoid any interference from nonspecific factors in the patients’ serum, 200 μL of the lysate was mixed with 20 mL of serum, which had been diluted 260 times and incubated overnight. The XL1-Blue MRF’ *E. coli* were then transfected with 200 μL of CNN2-λ-ZAP phage and an equal volume of phage-free solution, and then added to 6 mL of preheated top agar medium containing tetracycline. The mixture was spread onto preheated LB solid medium and incubated at 37 °C until plaques were visible. The plaques were blotted with a nitrocellulose (NC) membrane, and then the membrane was removed after incubation for 6 h. The NC membrane was immersed with PBS, blocked with 5% skim milk, shaken slowly for 2 h, and eluted with PBST for 10 min 3 times. The NC membrane was cut into several parts and numbered. Each NC membrane was immersed in the serum of patients diagnosed with HCC, gastric cancer, lung cancer, rectal cancer, hepatitis, and cirrhosis, and normal tissue, then diluted 260 times with 200 μL lysate for 2 h, and eluted with PBST for 10 min 3 times. After washing, the NC membrane was immersed in 1:10,000 alkaline phosphatase (AP)-linked goat anti-human IgG, incubated at room temperature with gentle shaking for 2 h, and eluted with PBST for 10 min 3 times. After washing the membrane, the color reaction was carried out with BCIP/NBT.

### 4.4. Construction and Purification of CNN2 Recombinant Protein

The CNN2-λ-ZAP phage previously constructed by our research group was used as the template to amplify the CNN2 gene by PCR. The primer sequence was as follows: forward: 5′-ATACATATGCACCATCATCATCATCATTCCCCGACGGTGGCGGCCGCT-3′; reverse: 5′-AGTGTCGACTCAGGGACCTCCCCCGGGTCCGGGCA-3′ (synthesized by Shanghai Bioengineering Co., Ltd., Shanghai, China). After PCR amplification, the DNA fragment and pCold III plasmid (3363, Takara Bio, Beijing, China) were digested by NdeI and SalI restriction endonuclease, after agarose gel electrophoresis, the gel was recovered and purified. Ligase linked the gene fragment with the plasmid and transformed the competent cell *DH5α*, and then positive clones were screened. The recombinant plasmid was extracted, then identified by double enzyme digestion, and sent to Shanghai Sangong Co., Ltd. (Shanghai, China) for sequencing. The correctly constructed recombinant expression plasmid (pColdIII-CNN2) was selected and transformed into *E. coli*. BL21 (DE3)pLysS (CD70102, TransGen Biotech, Beijing, China) competent cells, and positive bacterial clones were screened. The bacterial liquid was collected, and the supernatant and precipitate were taken for SDS-PAGE identification after the bacteria were broken. The gene engineering protein His-CNN2 was purified and identified by chromatography.

### 4.5. Indirect ELISA

We added 10 μg/mL of purified CNN2 recombinant protein to the ELISA plate at a concentration of 100 μL/well, and it was coated overnight at 4 °C. After washing, the plates were blocked with 0.5% bovine serum albumin (BSA) (A8010, Solabor Bioengineering Co., Ltd., Beijing, China) at 37 °C for 1 h. The serum of HCC, hepatitis B virus, liver cirrhosis, and normal tissue was diluted 1024 times with PBST, then 100 μL/well was added to the ELISA plate, and it was incubated at 37 °C for 1 h. Biotin-goat anti-human IgG (H + L) antibody (K1218, APExBIO, Shanghai, Beijing) was diluted 20,000 times, 100 μL/well was added to the ELISA plate, and it was incubated at 37 °C for 1 h. Streptavidin-HRP was diluted 9000 times, then 100 μL/well was added to the ELISA plate, and it was incubated at 37 °C for 1 h. Then, tetramethylbenzidine chromogenic solution (TMB) (IT0250, Solarbio Bioengineering Co., Ltd., Beijing, China) was added to the ELISA plate, with 100 μL/well, and it was incubated away from light for 15 min. The reaction was terminated by adding 2 M of concentrated sulfuric acid. The absorbance at 450 nm was measured by a microplate reader.

### 4.6. In Situ RT-PCR Analysis

Tissue sections (4 μm) were deparaffinized, digested by pepsin for 45 min, incubated overnight at 37 °C with H_2_O_2_-TBS, digested by protease K for 45 min, 95 °C for 2 min to inactivate protease K, and then treated by DNase at 37 °C for 1 h. RT-PCR reaction system (Hangzhou Bioer Technology Co., Ltd., Hangzhou, Zhejiang, China), including 1 mmol/L Digox-dUTP (100921, Beijing Zhongshan Jinqiao Biotechnology Co., Ltd., Beijing, China), was added. A PCR reaction was carried out as pre-denaturation at 95 °C for 3 min, and then 25 cycles (95 °C for 30 s, 44 °C for 1 min, 72 °C for 45 s) were performed for amplification. The primer sequence was as follows: forward: 5′-GTGGCTGAAGGATGGAACT-3′; reverse: 5′-TGTGTTCTGGAGGCTGATG-3′ (Shanghai Jierui Bioengineering Co., Ltd., Shanghai, China). Mouse anti-Digox IgG-AP (1:300) (DS0004, Beijing Zhongsan Jinqiao Biotechnology Co., Ltd., Beijing, China) was added and reacted with BCIP/NBT at room temperature for 30 min, and then it was observed and photographed.

### 4.7. Quantitative Real-Time PCR

The total RNA was obtained from the cultivated cells using TRIzol (R0016, Beyotime, Shanghai, China). The cDNA was synthesized using PrimeScript™ RT Kit with gDNA Eraser reagent (RR047Q, Takara Bio, Beijing, China). qRT-PCR amplification was performed with the SYBR Primix EX TaqTMII Real-Time PCR Kit (DRR041A, Takara Bio, Beijing, China). The primers used were as follows: CNN2: 5′-GGTCAAGGCCATATCCCAATAC-3′ (sense) and 5′-GGCATAGAAACCACAAACTGCTC-3′ (anti-sense), GAPDH: 5′-TGCACCACCAACTGCTTAGC-3′ (sense) and 5′-GGCATGGACTGTGGTCATGAG-3′ (anti-sense). The qRT-PCR was conducted with the following conditions: 95 °C for 2 min, followed by 40 cycles (95 °C for 15 s, 55 °C for 30 s, and 72 °C for 30 s). The relative expression of CNN2 mRNA in each group of samples was analyzed by the 2^−ΔΔCt^ method.

### 4.8. Immunohistochemistry

Paraffin sections were dewaxed and hydrated. Sections were retrieved by citrate buffer and incubated with 3% H_2_O_2_ at room temperature for 15 min. Goat serum was used to block nonspecific binding sites for 30 min and diluted rabbit anti-human CNN2 polyclonal antibody (1:200) was added overnight at 4 °C, followed by horseradish peroxidase-labeled anti-rabbit secondary antibody at 37 °C for 40 min. Slides were stained by diaminobenzidine (DAB) (ZLI9018, ZSGB-BIO, Beijing, China) and hematoxylin, then mounted by neutral balata. Pictures were taken by 200× magnification light microscope (MSC-B203T, Olympus, Beijing, China). Known positive HCC tissue sections were used as a positive control, and PBS was used instead of primary antibody as a blank control. Images were taken with a 200× magnification light microscope. The average optical density was calculated by image-Pro Plus 6.0 Image analysis software (version 6.0, Media Cybernetics, Rockville, MD, USA).

### 4.9. Western Blot

The cells were harvested, and the total protein was extracted and quantified with a BCA kit (P0010S, Beyotime, Shanghai, China). Each sample was mixed with loading buffer and bathed at 100 °C for 5 min. Then, 30 μg of the samples was loaded and separated into 12% SDS-PAGE gels (P0053A, Beyotime, Shanghai, China). The gels were then transferred to a polyvinylidene difluoride (PVDF) (1620184, Bio-Rad, Hercules, CA, USA) membrane at 100 V voltage. After sealing the non-specific binding sites with 5% skim milk, the PVDF membrane was incubated with primary antibody (SC136987, Santa, CA, USA) at 4 °C overnight. Then, horseradish peroxidase (HRP)—conjugated secondary antibody (HS201, TransGen Biotech, Beijing, China) was incubated. The PVDF film was developed according to the electrochemiluminescence (ECL) kit (P1010, APPLYGEN, Beijing, China) instructions. The gray value of the protein was analyzed by Image J software (Image J 1.8.0, National Institute of Health, Bethesda, MD, USA).

### 4.10. Construction of CNN2 Silencing Cell Lines

Four CNN2-siRNA sequences were designed. The siRNA gene of CNN2 was cloned into a pGPU6 expression vector containing GFP and Neo, and the RNAi eukaryotic plasmid expression vector pGPU6/GFP/Neo-CNN2-siRNA was constructed by Shanghai Gma Pharmaceutical. HepG2 cells (3~4 × 10^5^/mL) were inoculated in 24-well plates for culture (without penicillin and streptomycin). Plasmid transfection was performed after a cell confluence of 80–90% was reached, and the cell culture medium was changed to serum-free DMEM 4 h before transfection. Lipofectamine 2000 and siRNA plasmid were mixed into appropriate proportions, and the mixture was added to cultured HepG2 cells. After 6 h of culture, the medium containing 10% FBS was replaced. The cells were continuously cultured in DMEM containing 600 μg/mL G418. After 8 days, the cells in the untransfected group died completely, while a few cells in the transfected group survived. The cells were replaced with DMEM containing 300 μg/mL G418, and transferred to a 6-well plate when the cells became full of a 24-well plate, and 1.8 mL DMEM complete medium was added. When the cells were at a confluence of 100%, they were transferred to a 25 cm^2^ cell culture flask to detect their expression of CNN2 mRNA by real-time fluorescence quantitative PCR.

### 4.11. Cell Migration and Invasion Assay

A total of 1 × 10^6^ transfected cells and their control cells were inoculated in 6-well plates for routine culture for 48 h, and 10 μL pipette tips were used to scratch vertically in the wells, with 3 columns per well. After washing, pictures of the cells were taken and recorded. The migration status of cells at the scratched area was observed 24 h and 48 h after scraping, and the analysis was performed by Image J software (Image J 1.8.0, National Institute of Health, Bethesda, MD, USA), and repeated three times.

Matrigel gel (356234, BD, Franklin Lakes, NJ, USA) was diluted with serum-free DMEM (1:8) and evenly spread in a Transwell (8 μm aperture; Millipore) at the bottom of the chamber until the gelatin became solid. The cells were diluted with serum-free DMEM to 1.5 × 10^5^ cells/mL, and 200 μL cell suspension was added to the upper chamber. Then, 800 μL DMEM containing 10% FBS was added to the lower chamber, and it was incubated at 37 °C, 5% CO_2_ for 24 h. The medium was discarded, and the cells on the inner surface of the membrane were swabbed with cotton buds. The cells on the lower surface of membrane were dyed with 0.1% crystal violet for 10 min and then washed twice using ddH_2_O. Five fields (200×) were randomly selected under the microscope and counted, and the average value was taken.

### 4.12. Statistical Analysis

The experimental data were statistically analyzed using IBM SPSS software (version 22.0, IBM Corporation, Chicago, IL, USA) and plotted by GraphPad Prism 5 (GraphPad Prism Software Inc., San Diego, CA, USA) software. The measurement data were expressed as the mean ± standard deviation ($\bar{x}$ ± s), and the comparison between the two groups was performed by a *t*-test. Single-factor analysis of variance (one-way ANOVA) was used for comparison between groups conforming to the normal distribution and homogeneity of variance, and an LSD-*t* test was used for pound-wise comparison. The counted data were expressed by frequency and analyzed by *χ*^2^. Differences were statistically significant at *p* < 0.05.

## 5. Conclusions

This study demonstrates that CNN2 exhibited high expression levels in the serum, liver cancer cells, and tumor tissues of HCC patients, with significantly increased levels in metastatic HCC. The recombinant CNN2 protein was able to effectively detect HCC using indirect ELISA, while the down-regulation of CNN2 expression significantly reduced the migration and invasion of HCC cells. Therefore, CNN2 is considered an important protein related to HCC, with potential uses as a biomarker for the serological diagnosis of HCC, early risk prediction of HCC metastasis, and as a reference for optimizing HCC treatment regimens and improving patient survival rates. Additionally, CNN2 is a potential molecular target for HCC therapy, providing new avenues for tumor treatment.

## Figures and Tables

**Table 1:** Positive rate and *χ*^2^ test results of CNN2 antibody in serum of various patients and normal people.

Diseases	Case (*n*)	Positive Case (*n*)	Negative Case (*n*)	Positive Rate (%)	*p* Value
HCC *	31	17	14	54.8	<0.05
Gastric cancer	31	2	29	6.5	>0.05
Lung cancer	31	1	30	3.2	>0.05
Rectal cancer	31	3	28	9.7	>0.05
Hepatitis	31	1	30	3.2	>0.05
Liver cirrhosis	31	1	30	3.2	>0.05
Normal	32	1	31	3.1	>0.05

* HCC and other diseases were tested by pairwise *χ*^2^ test, *p* < 0.05.

## Data Availability

Data sets used or analyzed during the current study can be obtained from the corresponding authors upon reasonable request.

## Supplementary Materials

The supporting information can be download at: https://www.mdpi.com/article/10.3390/ijms24129864/s1.
