# Supplementary material for: The Role of H2-Calponin Antigen in Cancer Metastasis: Presence of Autoantibodies in Liver Cancer Patients
Source: Int J Mol Sci. 2023 Jun 7;24(12):9864. doi: 10.3390/ijms24129864 (PMC10298237; doi:10.3390/ijms24129864)
Supplement: Supplementary file 1 [file ijms-24-09864-s001.zip › ijms-2271955-supplementary.pdf]

Table S1. Relationship between clinical diagnosis and positive CNN2 antibody in serum.

Group	Positive Case	Negative Case	Positive rate (%)	χ^2	p
**Age**				1.478	0.224
⩾50	6	8	42.9		
⩽50	11	6	64.7		
**AFP**				1.873	0.171
Positive	8	10	44.4		
Negative	9	4	69.2		
**AFP content**				0.064	0.800
⩾400 ng/mL	3	2	60.0		
⩽400 ng/mL	14	12	53.8		
**BCLC Staging**				2.937	0.230
I	1	4	20		
II	10	6	62.5		
III	6	4	60		
**GGT content**				0.606	0.436
⩾50 U/L	12	8	60.0		
⩽50 U/L	5	6	45.5		

Table S2. Relationship between clinical diagnosis and positive of CNN2 antibody in serum by indirect ELISA.

Groups		Positive	Number	Positive Rate (%)	χ^2	p
**Age**	≥50	10	68	14.71	3.774	0.052
	≤50	16	55	29.09		
**Sex**	male	22	106	20.75	0.068	0.795
	female	4	17	23.53		
**AFP**	≥400	6	47	12.77	3.753	0.053
	≤400	20	72	27.78		
**BCLC**	I	7	46	15.22	1.587	0.452
	II	9	35	25.71		
	III	10	42	23.81		
**Cirrhosis or not**	yes	14	53	26.42	1.556	0.212
	no	12	70	17.14		
**ALT**	≥40	10	61	16.39	1.76	0.185
	≤40	16	61	26.63		
**AST**	≥40	16	70	22.86	0.234	0.629
	≤40	10	52	19.23		
**HBsAg**	+	24	107	22.43	0.823	0.364
	_	2	16	12.50		
**Tumor size**	<3 cm	2	12	16.67	0.171	0.679
	≥3 cm	24	110	21.82		

Table S3. Relationship between clinical diagnosis and expression of CNN2 protein in HCC.

Group	Number	Positive	Positive Rate (%)	χ^2	*p*
Gender				0.101	0.75
Man	41	21	51.22%		
Woman	13	6	46.15%		
Age					0.77
≤50	37	19	51.35%		
>50	17	8	47.06%		
BCLC Staging					0.537
I–II	34	18	52.94%		
III–IV	20	9	45.00%		
HCC with metastasis					0.028 *
Yes	30	19	63.33%		
No	24	8	33.33%		

* $p < 0.05$.
